# Conflict-Related Breakdowns in Routine Medical Care - Longitudinal Outcomes for Civilians with Pre-Existing Cognitive Impairment

**DOI:** 10.14336/AD.2024.0432

**Published:** 2025-05-31

**Authors:** Zorian Radomyslsky, Sara Kivity, Yaniv Alon, Mor Saban

**Affiliations:** ^1^Maccabi healthcare services, Tel Aviv-Jaffa, 6812509, Israel.; ^2^Department of Health system management, Ariel University, Ariel 40700, Israel.; ^3^Nursing Department, The Stanley Steyer School of Health Professions, Faculty of Medical and Health Sciences, Tel Aviv University, Tel Aviv, Ramat Aviv, 69978, Israel.

**Keywords:** dementia, armed conflict, healthcare disruption, vulnerable populations, longitudinal study, healthcare utilization, machine learning, health policy

## Abstract

Individuals with dementia face increased vulnerability during crises like armed conflicts. However, little is known about how conflicts affect dementia care delivery and patients' health. We conducted a longitudinal cohort study using medical record data. The study included 23,733 adults aged≥65 years with a diagnosis of dementia and 249,749 matched adults without dementia. Data were collected at baseline (March-October 2023), and two follow-up timepoints (December 2023 and February 2024), bracketing an armed conflict between Israel and Palestinian militant groups that began on October 7, 2023. We compared changes over time in clinical characteristics, medication use, healthcare utilization, costs between groups. Dementia prevalence was stable, but psychotropic medication use declined more sharply in those with dementia. Rates of depression diagnoses fell, and obesity rose in both groups. Healthcare utilization decreased substantially post-conflict, with fewer outpatient visits, hospitalizations, and emergency visits. Cost divergence between groups also increased over time. Machine learning identified shifting clusters of service users from high to mainly low users’ post-conflict. The conflict severely disrupted routine dementia care and altered health behaviors. Flexible service delivery and access promotion strategies are needed to support vulnerable populations like people with dementia during crises.

## INTRODUCTION

Dementia, a term encompassing a group of symptoms affecting memory, thinking, and social abilities, is not a specific disease but a manifestation of several diseases, with Alzheimer’s being the most common [1,2]. It is a significant health concern, with an estimated 6.9 million American adults diagnosed with dementia, including mild cognitive impairment, in 2024 [3].

The impact of dementia on patients is profound, significantly affecting their ability to perform daily functions and overall quality of life. As the illness progresses, symptoms worsen, and most individuals with dementia will eventually require assistance with daily activities [1,2, 4].

In times of war or emergency crises, the challenges faced by individuals with dementia are amplified. The disruption of routine, increased stress, and potential displacement can exacerbate dementia symptoms. Access to regular medical care and support services may be compromised, further jeopardizing their health and safety. Therefore, it is crucial to consider the needs of this vulnerable population in emergency planning and response strategies to mitigate adverse outcomes [5,6]. Past studies indicate that dementia patients are especially at-risk during crisis situations such as conflicts or natural disasters [7]. For instance, a post-Hurricane Katrina study revealed that dementia patients in nursing homes experienced higher hospitalization rates, medication errors, and behavioral deterioration compared to those without dementia [8]. Disruptions to their regular routines and the absence of consistent caregivers were identified as contributing factors [9]. Similarly, war veterans with dementia showed increased anxiety and agitation when exposed to trauma news coverage of conflicts. Interruptions to in-person care services have also been linked to cognitive decline and impaired daily task performance among dementia patients [10].

The ongoing conflict between Israel and the Palestinian militant group Hamas, which began on October 7th, 2023, has led to widespread casualties and destruction [11]. The unexpected attack by Hamas and other militant groups in Southern Israel resulted in the abduction of over 230 individuals, including Israeli and foreign nationals, who were taken to the Gaza Strip. This situation further compromised the sense of security for the region’s vulnerable elderly population, who were already facing risks from the armed conflict.

The outbreak of conflict severely disrupted the operation of essential dementia care services. Healthcare workers found it increasingly difficult to safely access patients for in-person checkups and care due to security concerns and limited transportation through conflict areas. Supply chains were also affected, making it challenging to reliably deliver medications, medical supplies, and nutritional provisions to patients. The sudden shortage of nursing home staff placed additional pressure on the remaining caregivers.

In light of the increased vulnerability of individuals living with dementia, the risk of neglected medical needs or disrupted care is significant. Within the Israeli healthcare landscape, a substantial portion of this population relies on specialized dementia units within nursing homes or in-home care programs facilitated by health maintenance organizations [12].

In response to the complex healthcare needs of individuals with dementia, it becomes crucial to highlight the significant mobilization of the healthcare system during periods of conflict in Israel. Maccabi Healthcare Services (MHS), a leading health management organization, plays a crucial role in spearheading initiatives to address the unique challenges faced by this population.

MHS’s response is multifaceted, including the establishment of unified clinics strategically located in hotels. These clinics serve as essential hubs, providing critical services to a diverse range of populations affected by the conflict. Concurrently, mobile clinics are dispatched to evacuation sites, ensuring the provision of necessary medical support to those in need.

This comparative study delves into the unique examination of the health status of patients with dementia amidst the ongoing Israel-Hamas war. The research focuses on understanding the impact of the conflict on clinical variables, medication usage, and overall health status in both the Study Group (individuals diagnosed with dementia) and the Control Group (individuals without a dementia diagnosis). Additionally, we explore the unique aspects of health services utilization, including Emergency Room (ER) visits, hospitalizations, outpatient appointments, telehealth consultations, and psychological support, all within the challenging context of an active war zone.

## METHODS

We conducted a prospective cohort study of older adults living in the community and insured under Maccabi Healthcare Services (MHS), one of the largest health management organizations in Israel. In MHS, there are over 300,000 insured individuals aged 65 and above. In 2019, MHS established the Cognitive Disorders Registry, which allows for the tracking of patients with cognitive decline in various clinical, therapeutic, managerial, and supportive arenas. Approximately 25,000 insured individuals are included in this database. Some are defined as being in the pre-dementia stage with mild cognitive impairment (MCI), while others suffer from rare dementia.

In our study, we used the Clinical Dementia Rating (CDR) scale to assess the severity of cognitive impairment in our participants. The CDR is a widely used tool for assessing the severity of dementia and is based on a semi-structured interview with both the patient and an informant, such as a family member or caregiver [4].

The CDR scale ranges from 0 to 3, with 0 indicating no cognitive impairment, 0.5 indicating very mild cognitive impairment, 1 indicating mild dementia, 2 indicating moderate dementia, and 3 indicating severe dementia. In our study, we used a CDR score of 0.5 or greater to identify participants with dementia. To differentiate mild cognitive impairment (MCI) from dementia, we used the criteria established by Petersen et al. (1999) [13], which define MCI as a cognitive decline that is greater than expected for a person's age and education level, but does not significantly interfere with their daily activities or meet the criteria for dementia. In our study, participants with a CDR score of 0.5 were classified as having dementia, while those with a CDR score of less than 0.5 were classified as having MCI or no cognitive impairment.

### Data Collection

Data collection was conducted over three distinct time points to capture a comprehensive snapshot of participant health and behaviors both before and after a significant event. The initial data gathering phase, referred to as the baseline, spanned from March 01 to October 07, 2023. This period provided a pre-event overview of participant conditions and behaviors. Following this, two subsequent follow-up collections were undertaken: the first on December 31, 2023, and the second on February 27, 2024, extending the observation window and enriching the longitudinal depth of the study. The collection process for each participant covered an extensive array of variables, categorized into three main groups, (1) This included data on age and gender (2) Clinical Characteristics: Participants were assessed for a range of clinical variables, such as the presence of dementia, usage and prescription of antipsychotic and antidepressant medications, depression diagnosis, bone fractures, blood pressure issues, chronic obstructive pulmonary disease (COPD), diabetes mellitus (DM), immunosuppression, and obesity. (3) Health Services Utilization: This encompassed metrics on healthcare service engagement, including visits to ERs, hospitalization durations, outpatient visits to geriatric and family physicians, telehealth consultations, social worker and psychologist sessions, and receipt of home-based treatments.

### Data Analysis

The longitudinal study adopted a multi-pronged analytical methodology to examine the interactions between the Study Group—consisting of individuals diagnosed with dementia—and the Control Group—comprising those without such a diagnosis—within the elderly population aged 65 and older. The analysis spanned three time points: baseline, first follow-up, and second follow-up, employing statistical techniques customized for each measured variable.

To evaluate the distribution of sex across the study and control groups, Chi-square tests were employed. For continuous variables such as age and cost, independent samples T-tests were used to compare mean differences between the study and control groups at each time point. To quantify the effect size of cost differences across the baseline, follow-up 1, and follow-up 2 periods, Cohen’s d statistic was calculated. For the analysis of binary outcome variables across repeated measures, Generalized Estimating Equations (GEE) were utilized. This method allowed us to examine the correlation between baseline conditions and outcomes at subsequent follow-up intervals, accounting for within-subject correlation. For quantitative variables, we employed Mixed Model Analysis of Variance (ANOVA) to assess both fixed effects, such as temporal factors and group membership, and random effects, including individual variations. Association among time, depression diagnosis, antidepressant adherence, and immune suppression, was measured via Mixed Linear Model (MLM). Additionally, to detect patterns in healthcare utilization among participants, the Density-Based Spatial Clustering of Applications with Noise (DBSCAN) algorithm was employed. Prior to clustering, data normalization was performed to ensure accurate reflection of variations. Post-clustering, a RandomForestClassifier was applied to extend the model’s applicability to the control group, facilitating a thorough comparison between groups. Data analysis was performed using Python 3.0, and a significance threshold of *p* < 0.05 was applied for all statistical analyses.

### Ethical Considerations

The research protocol received approval from the Institutional Human Subjects Ethics Committee (0075-24-MHS) of the concerned medical facility. The Institutional Review Board granted a waiver for written informed consent. All procedures were conducted in accordance with the ethical standards of the institutional and national research committees, adhering to national ethical guidelines.

## RESULTS

In this study, we defined two distinct cohorts: the Study Group, comprised of individuals diagnosed with dementia, and the Control Group, composed of individuals without such a diagnosis, both focusing on the elderly population aged 65 and above.

Data for each group were collected across three-time intervals: the baseline period, which commenced on October 6, 2023, and two subsequent follow-up points on December 31, 2023, and February 27, 2024. To ensure cohort homogeneity, certain individuals were excluded based on the criteria outlined in Supplementary Figure 1. First, participants lacking data across all time frames were omitted to facilitate more effective analysis of post-October 7th effects, including those who had passed away before this date. Subsequently, the Study Group saw a reduction of 10,139 members, while the Control Group witnessed a decrease of 58,200 members. Further refining the age profiles of both cohorts by eliminating age-related anomalies led to the removal of an additional 2,730 members from the Study Group and 10,652 from the Control Group. Consequently, the Study Group's final dataset comprised 23,733 members, and the Control Group consisted of 249,749 members. In the research of remaining populations across both cohorts, as outlined in Table 1, our analysis revealed several notable insights. The study employed Generalized Estimating Equations (GEE) (Supplementary Table 1) to determine the association between baseline alterations and subsequent observations at follow-up intervals.

Table 1 delineates the prevalence of various health conditions within elderly cohorts, conducting a temporal comparative analysis between groups diagnosed with dementia and those devoid of such a diagnosis. The prevalence of dementia remained static across both cohorts, with approximately 21% of the study group receiving diagnoses consistently across all periods. In parallel, the control group witnessed no emergent cases. A closer examination of comorbidities between the first two evaluation periods revealed minimal fluctuations. Such stability was anticipated, particularly considering the events of October 7th, 2023, which were hypothesized to impact psychological rather than physical health domains. One noteworthy observation showed that the incidence of hip fractures in the study group had a significant reduction of 8% (N=1,904, p<0.001), alongside a decline by 5.34% (N=13,338, p<0.001) in the control group. This trend potentially reflects a decreased tendency among the elderly to engage in outdoor activities amid wartime risks. This hesitation persisted into the follow-up 2 period, indicating a continued aversion to outdoor pursuits among older adults in both cohorts. In addition, the follow-up 2 period marked a notable increase in obesity rates within both populations. Specifically, a significant surge from baseline to follow-up 2 by 24.18% (N = 5,737, p<0.001) was observed in the study group, with an 18.92% increase (N = 47,255, p<0.001) in the control cohort. This pattern suggests a cumulative effect of reduced physical activity over time, contributing to a pronounced rise in obesity rates by the second follow-up. However, this increase was absent between the baseline and the first follow-up, underscoring the gradual impact of sedentary lifestyles. It is worth noting that diabetes rates remained consistent between the baseline and follow-up 1 periods but showed a slight increase in follow-up 2 period. This suggests that the growing prevalence of obesity may be beginning to impact the rate of diabetes in individuals in both groups. An unanticipated result was the decline of depression diagnoses among dementia patients within the study group, which decreased by 8.47% (N=2047, p<0.001) approximately three months post-event. This result was unexpected given the anticipated emotional strain on the Israeli population. contrary, the control group exhibited a marginal reduction in depression diagnoses by 2.35% (N= 5,890, p<0.001). These results were similar in follow-up 2 period, both for study and control group.

**Table 1:** Health Condition Prevalence in Elderly Populations: Comparative Analysis of Dementia and Non-Dementia Groups Over Time, after the events of October 7th, 2023.

	Group	Age	Total n	p value			
*Age, mean± std*	Study	78.63±6.2	23733	p<0.001			
		
	Control	72.45±5.7	249749				
		
*Sex, N (%)*	Study	M	8965 (37.77%)	p<0.001			
		**F**	14768 (62.23%)				
	Control	M	114709 (45.93%)				
		**F**	135040 (54.07%)				
**Condition**	**Group**	**Status**	**Baseline, N (%)**	**Follow-Up 1, N (%)**	**Follow-Up 2, N (%)**	**p value Follow-up 1 vs. Baseline**	**p value Follow-up 2 vs. Baseline**
*Dementia*	Study	Healthy	18752 (79.01%)	18752 (79.01%)	18725 (78.9%)	p <0.001	p <0.001
		**Suffer**	4981 (20.99%)	4981 (20.99%)	5008 (21.1%)	p <0.001	p <0.001
	Control	Healthy	249749 (100.0%)	249749 (100.0%)	249749 (100.0%)	p <0.001	p <0.001
		**Suffer**	0 (0.0%)	0 (0.0%)	0 (0.0%)	p <0.001	p <0.001
*Depression*	Study	Healthy	17070 (71.93%)	19066 (80.34%)	19177 (80.8%)	p <0.001	p <0.001
		**Suffer**	6663 (28.07%)	4667 (19.66%)	4556 (19.2%)	p <0.001	p <0.001
	Control	Healthy	227945 (91.27%)	233775 (93.6%)	233970 (93.68%)	p <0.001	p <0.001
		**Suffer**	21804 (8.73%)	15974 (6.4%)	15779 (6.32%)	p <0.001	p <0.001
*Blood pressure*	Study	Healthy	5478 (23.08%)	5460 (23.01%)	5468 (23.04%)	p <0.001	p <0.001
		**Suffer**	18255 (76.92%)	18273 (76.99%)	18265 (76.96%)	p <0.001	p <0.001
	Control	Healthy	90367 (36.18%)	90105 (36.08%)	90259 (36.14%)	p <0.001	p <0.001
		**Suffer**	159382 (63.82%)	159644 (63.92%)	159490 (63.86%)	p <0.001	p <0.001
*Chronic obstructive pulmonary disease (COPD)*	Study	Healthy	21400 (90.17%)	21396 (90.15%)	21391 (90.13%)	p <0.001	p <0.001
		**Suffer**	2333 (9.83%)	2337 (9.85%)	2342 (9.87%)	p <0.001	p <0.001
	Control	Healthy	230787 (92.41%)	230710 (92.38%)	230667 (92.36%)	p <0.001	p <0.001
		**Suffer**	18962 (7.59%)	19039 (7.62%)	19082 (7.64%)	p <0.001	p <0.001
*Diabetes mellitus (DM)*	Study	Healthy	14770 (62.23%)	14752 (62.16%)	14375 (60.57%)	p <0.001	p <0.001
		**Suffer**	8963 (37.77%)	8981 (37.84%)	9358 (39.43%)	p <0.001	p <0.001
	Control	Healthy	175608 (70.31%)	175318 (70.2%)	171845 (68.81%)	p <0.001	p <0.001
		**Suffer**	74141 (29.69%)	74431 (29.8%)	77904 (31.19%)	p <0.001	p <0.001
*Immune suppression*	Study	Healthy	19264 (81.17%)	19180 (80.82%)	21599 (91.01%)	p <0.001	p <0.001
		**Suffer**	4469 (18.83%)	4553 (19.18%)	2134 (8.99%)	p <0.001	p <0.001
	Control	Healthy	210810 (84.41%)	210085 (84.12%)	234046 (93.71%)	p <0.001	p <0.001
		**Suffer**	38939 (15.59%)	39664 (15.88%)	15703 (6.29%)	p <0.001	p <0.001
*Obesity*	Study	Healthy	16437 (69.26%)	16427 (69.22%)	10700 (45.08%)	p <0.001	p <0.001
		**Suffer**	7296 (30.74%)	7306 (30.78%)	13033 (54.92%)	p <0.001	p <0.001
	Control	Healthy	177254 (70.97%)	177090 (70.91%)	129999 (52.05%)	p <0.001	p <0.001
		**Suffer**	72495 (29.03%)	72659 (29.09%)	119750 (47.95%)	p <0.001	p <0.001
*Hip Fracture*	Study	Healthy	21263 (89.59%)	23167 (97.62%)	23196 (97.74%)	p <0.001	p <0.001
		**Suffer**	2470 (10.41%)	566 (2.38%)	537 (2.26%)	p <0.001	p <0.001
	Control	Healthy	231935 (92.87%)	245273 (98.21%)	245794 (98.42%)	p <0.001	p <0.001
		**Suffer**	17814 (7.13%)	4476 (1.79%)	3955 (1.58%)	p <0.001	p <0.001

The table presents descriptive statistics from a longitudinal study, comparing the prevalence of various health conditions between a group diagnosed with dementia (N=23,733) and a control group of elderly individuals without dementia (N=249,749), all aged over 65. Data were collected at baseline (prior to October 7, 2023) and at two subsequent follow-up points (December 31, 2023, and February 27, 2024). Age significance level was via T.test and sex via chi square. The monitored health conditions include depression, hypertension, chronic obstructive pulmonary disease (COPD), diabetes, immune suppression, obesity, and hip fractures. Statistical significance was assessed using Generalized Estimating Equations (GEE) analysis, comparing baseline data with follow-up 1 and follow-up 2 periods.

To understand trends in depression diagnoses, we conducted an analysis concentrating on patient adherence to prescribed medications (significant levels were tested and are presented in Supplementary Table 1. Our investigation focused on two main medication categories: antidepressants and antipsychotics. This analysis aimed to delineate the disparities between prescription rates and actual medication utilization across three distinct time periods.

**Figure 1.**
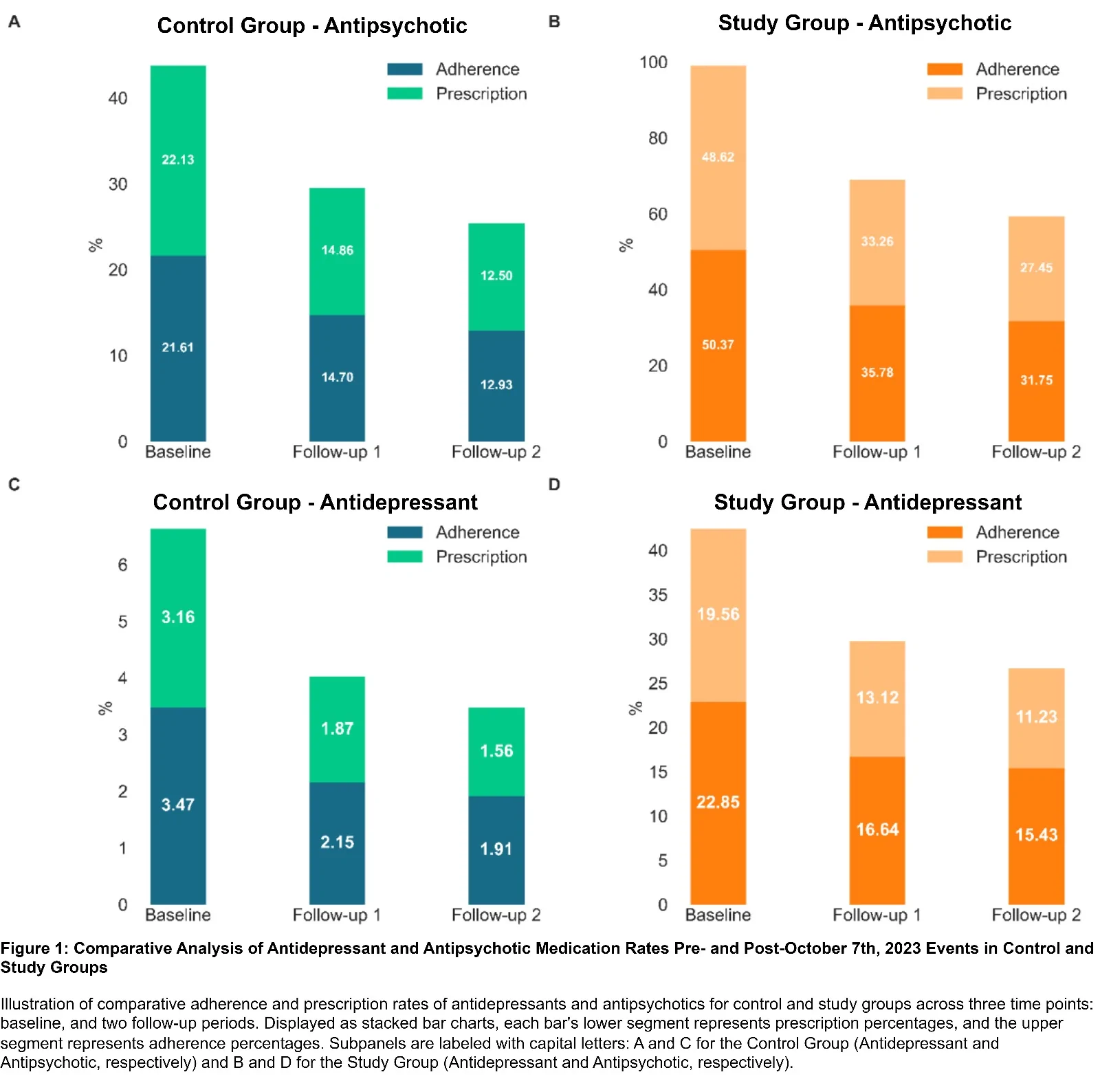
**The comparative adherence and prescription rates of antidepressants and antipsychotics for control and study groups across three time points: baseline, and two follow-up periods**. Displayed as stacked bar charts, each bar's lower segment represents prescription percentages, and the upper segment represents adherence percentages. Subpanels are labelled with capital letters: (**A**) and (**C**) for the Control Group (Antidepressant and Antipsychotic, respectively) and (**B**) and (**D**) for the Study Group (Antidepressant and Antipsychotic, respectively).

The results (Fig. 1) revealed a significant decline in antidepressant prescriptions within the control group, marking a 7.27% decrease (N=18,156, p<0.001) from the baseline to the first follow-up period, and an additional 2.36% decline leading into the second follow-up period. This accumulates to a reduction of 9.63% (N=24,050, p<0.001) from the baseline to the second follow-up period. Correspondingly, adherence to antidepressant medication within the control group mirrored this trend, showing a 6.91% decline (N=17,257, p<0.001) from the baseline to the first follow-up, with an overall decrease of 8.63% (N=21,553, p<0.001) by the second follow-up period. The study group exhibited a steeper decline in antidepressant prescriptions, with a 15.36% reduction (N=3,645, p<0.001) from the baseline to the first follow-up period, and an additional 5.81% decline leading to a total 21.17% decrease (N=5,024, p<0.001) by the second follow-up period. Adherence rates within the study group showed a similar pattern, with a 14.6% reduction (N=3,465, p<0.001) initially, culminating in an 18.95% decline (N=4,497, p<0.001) by the second follow-up. Antipsychotic medications, however, displayed a slower rate of decline in the control group, where both prescriptions and adherence levels were initially low. Within the study group, there was a significant decrease with a 6.44% reduction in prescriptions (N=1,528, p<0.001) and a 6.21% reduction in adherence (N=1,473, p<0.001) from baseline to the first follow-up period, followed by a subsequent slight decline, indicating a plateau in the reduction rate by the second follow-up period.

The decrease in prescription and subsequent decrease in adherence to antidepressants may account for the reduction in diagnosed immune suppression (see Table 1). The low adherence to antidepressants showed no significant change between the baseline period and the initial follow-up. However, the reduction in antidepressant adherence between the baseline and the second follow-up allowed for sufficient time for an accumulated change in diagnosed immune suppression. In both groups, there was a reduction of diagnoses around half of the bassline diagnosis: 9.84% (N=2,335, *p*<0.001) for the study group and 9.3% (N=23,236, *p*<0.001) for the control group, respectively. To investigate whether our data could reveal a connection between these findings, we employed Mixed Linear Model (MLM) regression analysis, which uncovered significant associations between depression diagnosis, antidepressant adherence, and the likelihood of immune suppression (Supplementary Table 2).

Specifically, individuals diagnosed with depression were found to have a slightly higher probability of immune suppression, indicated by a coefficient of 0.048 (*p*<0.001). Similarly, antidepressant adherence was associated with an increased probability of immune suppression, with a coefficient of 0.051 (*p*<0.001). Notably, the interaction between depression diagnosis and antidepressant adherence exhibited a negative coefficient of -0.045 (*p*<0.001), suggesting a moderating effect where the simultaneous presence of depression and antidepressant use does not elevate the risk of immune suppression as much as their independent effects might imply. The analysis did not identify a significant temporal effect on immune suppression or its variance across the groups, indicating a stable relationship over the observed periods. These different changes, both in medication compliance and diagnoses of depression, immune-suppression and rise of obesity, mandate a deeper exploration into healthcare service utilization.

The reduction in healthcare services, as detailed in Table 2, illustrates the significant impact of the events on October 7th, 2023, on the elderly population's willingness to seek healthcare. Across all services, there was a noticeable decline in healthcare utilization from the baseline period to the subsequent follow-up periods. Notably, inquiries to the ER decreased substantially, from 0.568±1.26 in the Study Group and 0.337±0.847 in the Control Group at baseline to 0.005±0.069 and 0.002±0.049, respectively, by the second follow-up. Hospitalization days and the number of hospitalizations followed a similar declining trend. For instance, hospitalization days in the Study Group reduced from 5.349±23.473 at baseline to 0.015±0.52 by the second follow-up. This pattern signifies a marked decrease in hospital stays, likely influenced by a combination of reduced healthcare seeking and possible shifts in hospital admission criteria during the studied period. Moreover, there was a noteworthy decline in frontal visits to both geriatric and family physicians. Initially, the Study Group had 0.586±1.362 geriatric physician visits and 15.039±12.74 family physician visits at baseline, which declined to 0.056±0.27 and 1.424±1.784, respectively, by the second follow-up. These figures indicate a significant reduction in direct physician consultations, further underscoring the elderly's reluctance or inability to seek routine medical advice during and after the events of October 7^th^, 2023. Phone appointments and social worker visits also saw reductions, though these were less dramatic compared to other services.

**Table 2:** Impact of October 7th Events with two follow-up periods, on Healthcare Service Utilization Among Elderly Populations.

Healthcare Services	Group	Baseline	Follow-Up 1	Follow-Up 2	*p* value Follow-up 1 vs. Baseline	*p* value Follow-up 2 vs. Baseline
Inquiries To the Er, mean±std	Study	0.568±1.26	0.021±0.152	0.005±0.069	p<0.001	p<0.001
	**Control**	0.337±0.847	0.012±0.12	0.002±0.049	p<0.001	p<0.001
Hospitalization Days, mean±std	Study	5.349±23.473	0.105±1.103	0.015±0.52	p<0.001	p<0.001
	**Control**	2.569±17.253	0.057±0.789	0.008±0.389	p<0.001	p<0.001
Hospitalization, mean±std	Study	0.939±1.845	0.028±0.196	0.004±0.065	p<0.001	p<0.001
	**Control**	0.592±1.452	0.019±0.162	0.003±0.057	p<0.001	p<0.001
Frontal Visits to Geriatric Physician Visits, mean±std	Study	0.586±1.362	0.074±0.332	0.056±0.27	p<0.001	p<0.001
	**Control**	0.056±0.484	0.008±0.111	0.007±0.102	p<0.001	p<0.001
Frontal Visits to Family Physician, mean±std	Study	15.039±12.74	1.754±2.132	1.424±1.784	p<0.001	p<0.001
	**Control**	11.83±10.513	1.471±1.856	1.243±1.595	p<0.001	p<0.001
Phone appointments with Geriatric Physician, mean±std	Study	0.141±1.287	0.031±0.327	0.02±0.247	p<0.001	p<0.001
	**Control**	0.013±0.436	0.003±0.114	1.243±1.595	p<0.001	p<0.001
Phone appointments with Family Physician, mean±std	Study	0.608±3.019	0.136±0.708	0.101±0.574	p<0.001	p<0.001
	**Control**	0.133±1.209	0.033±0.309	0.026±0.253	p<0.001	p<0.001
Social Worker Visits, mean±std	Study	1.057±2.89	0.16±0.658	0.121±0.552	p<0.001	p<0.001
	**Control**	0.284±1.682	0.046±0.39	0.038±0.34	p<0.001	p<0.001
Visit to the Psychologist, mean±std	Study	0.03±0.804	0.004±0.193	0.004±0.16	p<0.001	p<0.001
	**Control**	0.037±1.033	0.005±0.188	0.012±0.147	p<0.001	p<0.001
Eligible for Home Treatments, N (%)	Study	1982 (8.35%)	1822 (7.68%)	1879 (7.92%)	p<0.001	p<0.001
	**Control**	2376 (0.95%)	2376 (0.95%)	2163 (0.87%)	p<0.001	p<0.001

Descriptive analysis was conducted on the average utilization of various healthcare services, encompassing emergency room visits, hospitalization days, total hospitalizations, and consultations with geriatric and family doctors. Quantitative significance levels were determined using a Mixed-Effects Anova Model while binary significance was assessed using GEE analysis

To interpret the patterns of health service utilization, we employed the Density-Based Spatial Clustering of Applications with Noise (DBSCAN), an unsupervised machine learning algorithm, using the healthcare services described in Table 2 as features. Although our analysis is limited by the absence of data on socioeconomic status and geographic location, and thus we could not explore how variations in access to healthcare services based on these factors might have influenced our findings, this method allowed us to assign a healthcare usage score to each subject within both the study and control groups. This facilitated the identification of 'heavy users' across the baseline and follow-up periods and enabled us to recognize aggregate trends in usage.

**Figure 2.**
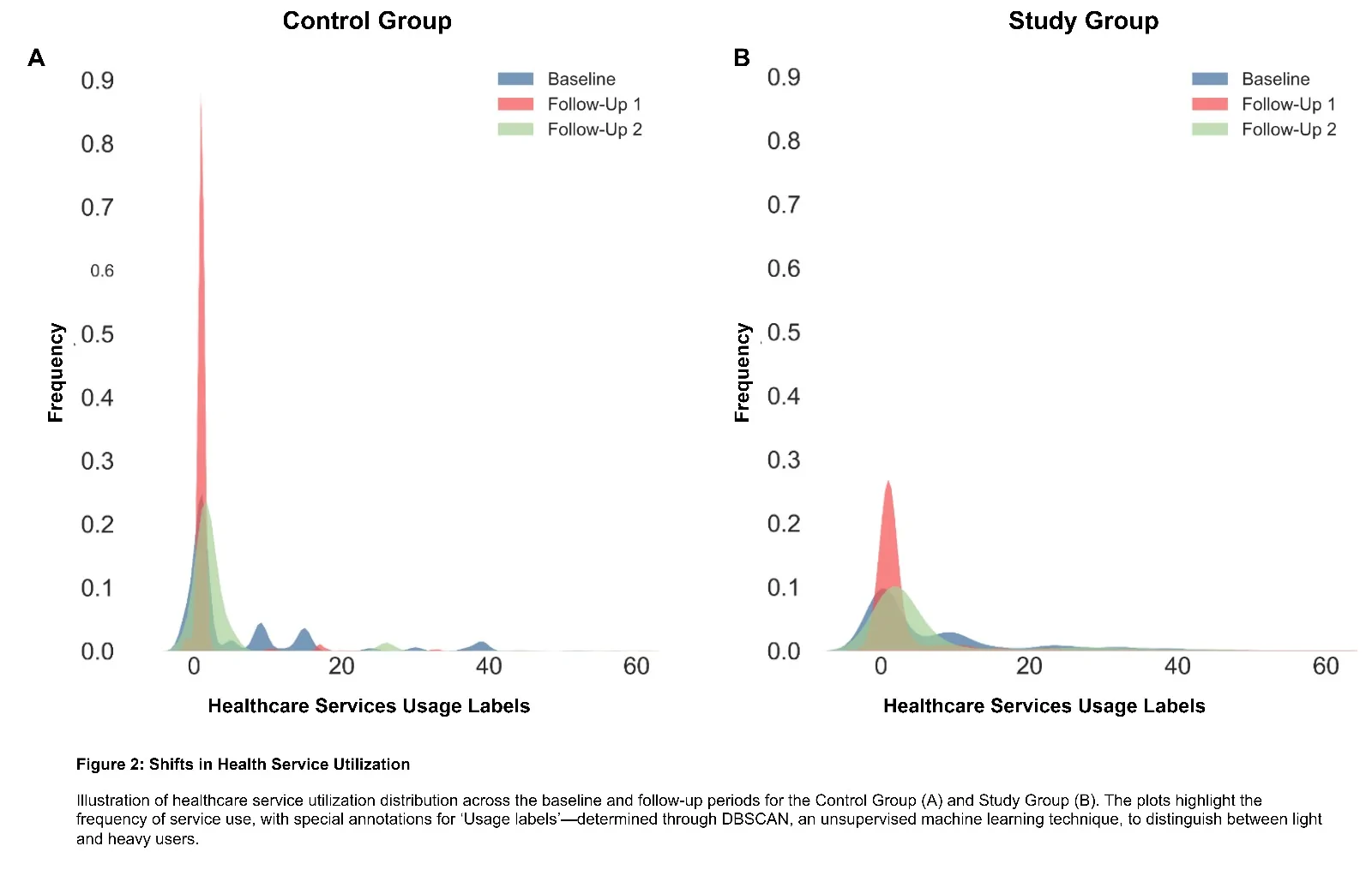
**The healthcare service utilization distribution across the baseline and follow-up periods for the Control Group (A) and Study Group (B)**. The plots highlight the frequency of service use, with special annotations for ‘Usage labels’—determined through DBSCAN, an unsupervised machine learning technique, to distinguish between light and heavy users.

The analysis of Figure 2 reveals a significant shift in healthcare service utilization among the elderly, with a transition from a broader distribution of heavy users during the baseline period to a narrower distribution, indicating a predominance of light users, in the first follow-up period. This change illustrates a marked decrease in healthcare engagement, potentially due to increased caution or accessibility challenges amidst the ongoing crisis. However, by the second follow-up, the distribution begins to widen again, suggesting a gradual return towards pre-crisis levels of healthcare utilization. This trend towards normalization could be interpreted as a positive sign of recovery and adaptation among the elderly population. Despite this seemingly positive trend towards increased healthcare engagement indicated by the shift back to a wider distribution in Figure 2, Table 2 presents a contrasting narrative of a continuous decline in healthcare service usage, such as reduced ER visits and hospitalizations.

**Figure 3.**
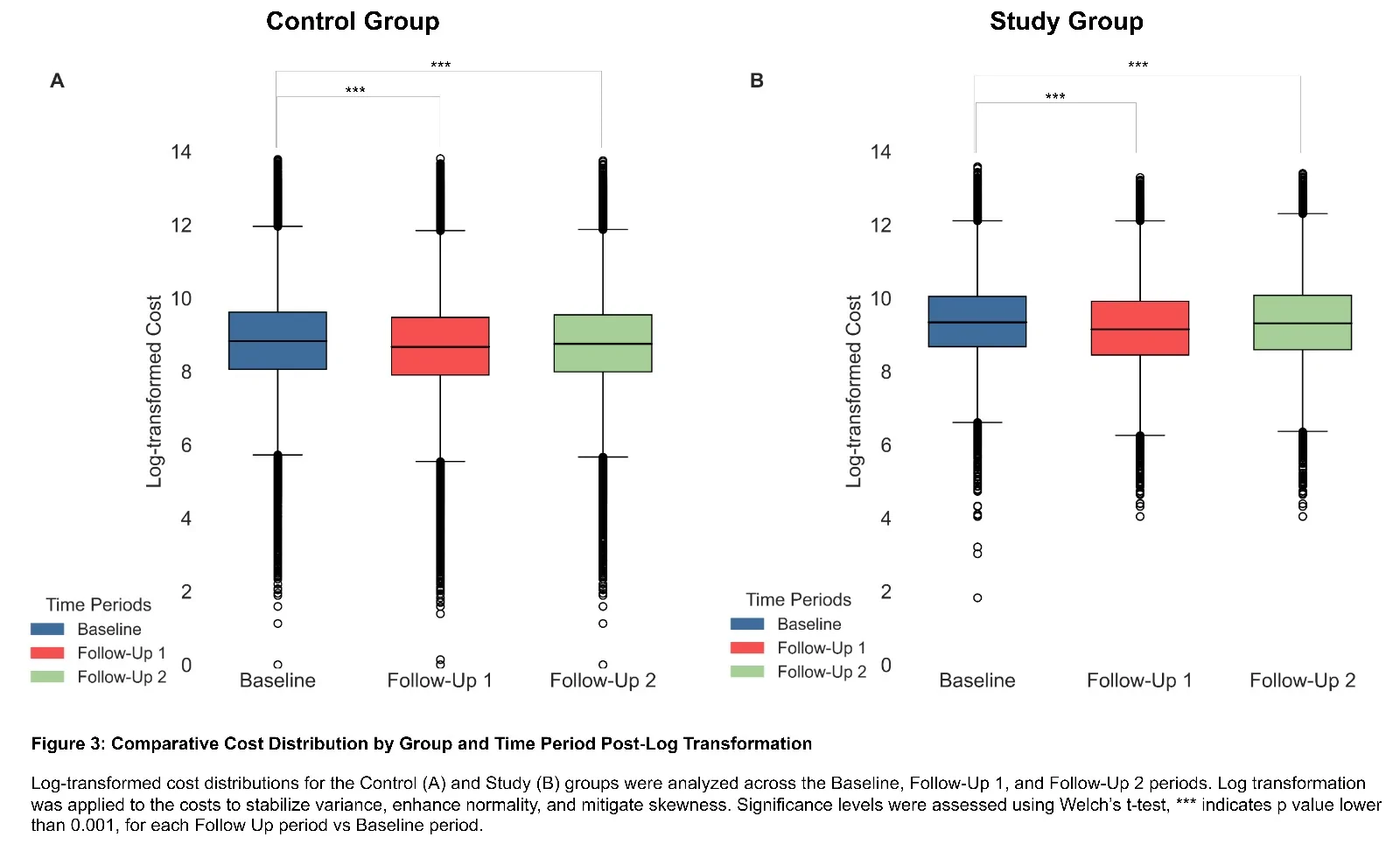
**The Log-transformed cost distributions for the Control (A) and Study (B) groups were analysed across the Baseline, Follow-Up 1, and Follow-Up 2 periods**. Log transformation was applied to the costs to stabilize variance, enhance normality, and mitigate skewness. Significance levels were assessed using Welch’s t-test, *** indicates p value lower than 0.001, for each Follow Up period vs Baseline period.

The observed shift in health service utilization is also evident in the change in costs. Figure 3 offers a detailed view of the comparative cost distribution between the Study and Control groups across three time periods: Baseline, Follow-Up 1, and Follow-Up 2, with costs undergoing log transformation to enhance normality and minimize variance and skewness. The statistical analysis, incorporating Cohen's d, reveals significant differences in cost dynamics across the time periods, indicating meaningful variations in healthcare spending. At Baseline, Cohen's d is calculated at 0.4, suggesting a moderate effect size in the cost differences between the groups. This effect size slightly decreases in Follow-Up 1 to 0.37, before increasing again in Follow-Up 2 to 0.44. With affinity to the patterns of healthcare utilization discussed previously, where a return to broader engagement was observed in Figure 2, the increasing cost in Follow-Up 2 as depicted in Figure 3 and corroborated by the rising Cohen's d value, could reflect either an increase in the volume of services used, the use of more expensive services, or both.

## DISCUSSION

The study examined the impact of the events on October 7th, 2023, on the health and healthcare utilization of two elderly cohorts: those diagnosed with dementia (Study Group) and those without such a diagnosis (Control Group). While the findings reveal significant changes in health conditions, medication adherence, and healthcare service utilization, it is important to note that the study's observational design precludes the establishment of causal relationships between the Israel-Hamas conflict and these changes among dementia patients.

The results of this study highlight the profound impact of the events on October 7th, 2023, on the health and healthcare utilization of the elderly population in Israel. Both the study group and the control group experienced notable shifts in health and healthcare patterns. The prevalence of dementia remained consistent across both cohorts, with no emergent cases in the control group. This stability was expected, given that the events of October 7th, 2023, were hypothesized to impact psychological rather than physical health domains. However, the study uncovered several notable health trends. For instance, there was a significant reduction in the incidence of hip fractures in both groups, potentially reflecting a decreased tendency among the elderly to engage in outdoor activities amid wartime risks. This trend persisted into the second follow-up period, indicating a continued aversion to outdoor pursuits among older adults in both cohorts [14].

Moreover, the second follow-up period marked a notable increase in obesity rates within both populations. This pattern suggests a cumulative effect of reduced physical activity over time, contributing to a pronounced rise in obesity rates by the second follow-up [15]. However, this increase was absent between the baseline and the first follow-up, underscoring the gradual impact of sedentary lifestyles [14]. It is worth noting that DM rates remained consistent between the baseline and follow-up 1 periods but showed a slight increase in the follow-up 2 period. This suggests that the growing prevalence of obesity may be beginning to impact the rate of DM in individuals in both groups.

An unanticipated result was the decline of depression diagnoses among dementia patients within the study group, which decreased by 8.47% approximately three months post-event. This result was unexpected given the anticipated emotional strain on the Israeli population. In contrast, the control group exhibited a marginal reduction in depression diagnoses by 2.35%. These results were similar in the follow-up 2 period, both for the study and control group.

To understand trends in depression diagnoses, we conducted an analysis focusing on patient adherence to prescribed medications. Our investigation focused on two main medication categories: antidepressants and antipsychotics. This analysis aimed to delineate the disparities between prescription rates and actual medication utilization across three distinct time periods [16]. The data reveal a significant decline in antidepressant prescriptions within both groups [17,18], with a steeper decline in the study group. Antipsychotic medications, however, displayed a slower rate of decline in the control group [19,20], where both prescriptions and adherence levels were initially low. Within the study group, there was a significant decrease with a subsequent slight decline, indicating a plateau in the reduction rate by the second follow-up period [21].

The decrease in prescription and subsequent decrease in adherence to antidepressants may account for the reduction in diagnosed immune suppression. The low adherence to antidepressants showed no significant change between the baseline period and the initial follow-up. However, the reduction in antidepressant adherence between the baseline and the second follow-up allowed for sufficient time for an accumulated change in diagnosed immune suppression. In both groups, there was a reduction of diagnoses around half of the baseline diagnosis [22]. To investigate whether our data could reveal a connection between these findings, we employed MLM regression analysis, which uncovered significant associations between depression diagnosis, antidepressant adherence, and the likelihood of immune suppression.

The events of October 7th, 2023, also had a significant impact on the elderly population’s healthcare-seeking behavior, as evidenced by the reduction in healthcare services utilization [23]. This trend was observed across all services, with the most notable decline in ER inquiries. The decrease in hospitalization days and the number of hospitalizations suggests a marked reduction in hospital stays, which could be attributed to a combination of reduced healthcare seeking and potential shifts in hospital admission criteria during the studied period [24]. The decline in frontal visits to both geriatric and family physicians underscore the elderly’s reluctance or inability to seek routine medical advice during and after the events. This is further evidenced by the significant reduction in direct physician consultations [24]. While phone appointments and social worker visits also saw reductions, these were less dramatic compared to other services. These findings highlight the need for strategies to encourage healthcare-seeking behavior among the elderly, particularly in the wake of significant events [25].

To interpret the patterns of health service utilization in the current study, we employed the DBSCAN, an unsupervised machine learning algorithm, using the health care services. This method allowed us to assign a healthcare usage score to each subject within both the study and control groups, thus facilitating the identification of ‘heavy users’ across the baseline and follow-up periods [26,27]. The analysis reveals a significant shift in healthcare service utilization among the elderly, with a notable transition from a broader distribution of heavy users during the baseline period to a narrower distribution, indicating a predominance of light users, in the first follow-up period. This change illustrates a marked decrease in healthcare engagement, potentially due to increased caution or accessibility challenges amidst the ongoing crisis.

However, by the second follow-up, the distribution begins to widen again, suggesting a gradual return towards pre-crisis levels of healthcare utilization. This trend towards normalization could be interpreted as a positive sign of recovery and adaptation among the elderly population. Despite this seemingly positive trend towards increased healthcare engagement indicated by the shift back to a wider distribution, Table 2 presents a contrasting narrative of a continuous decline in healthcare service usage, such as reduced ER visits and hospitalizations. The observed shift in health service utilization is also evident in the change in costs. The statistical analysis, incorporating Cohen’s d, reveals significant differences in cost dynamics across the time periods, indicating meaningful variations in healthcare spending. At Baseline, Cohen’s d is calculated at 0.4, suggesting a moderate effect size in the cost differences between the groups. This effect size slightly decreases in Follow-Up 1 to 0.37, before increasing again in Follow-Up 2 to 0.44. In juxtaposition to the patterns of healthcare utilization discussed previously, where a return to broader engagement was observed, the increasing cost in Follow-Up 2 could reflect either an increase in the volume of services used, the use of more expensive services, or both.

To provide a comprehensive understanding of the breakdowns in routine medical care for civilians with pre-existing cognitive impairment in conflict-affected settings, it is important to consider the broader effects of conflict on health and well-being. In this context, we refer to a recent survey conducted during the same conflict, aiming to investigate the self-reported effects of conflict on a population exposed to regional conflict, inclusive of individuals with various types of cognitive impairment [28].

The survey findings revealed a significant decrease in the proportion of Israelis who rated their health as excellent or very good, while the proportion perceiving their health status as moderate or poor almost doubled following the war. Additionally, the survey highlighted that 30% of Israelis with chronic illnesses reported a worsening of their condition due to the conflict. Disruptions to healthcare services were also evident, with 36% of respondents reporting the postponement or cancellation of doctor's appointments or medical tests. Furthermore, 17% of patients reported postponing or canceling important screening tests.

Although the survey did not specifically focus on dementia patients, it provides valuable insights into the broader effects of conflict on physical and psychological health in a conflict-affected community [28]. In conclusion, this study sheds light on the significant impact of the events on October 7th, 2023, on the health and healthcare utilization of the elderly population in Israel. The findings provide valuable insights into the effects of such events on healthcare systems and can inform future strategies to mitigate these impacts and improve the resilience of healthcare services in times of crisis. However, further research, utilizing more rigorous research designs, is needed to explore the long-term effects of these events on the health and wellbeing of the elderly population while considering potential confounders and multiple factors.

### Limitations

While our study offers crucial insights into dementia care amidst armed conflict, we acknowledge certain limitations that should be considered. One limitation is the focus primarily on the elderly population in Israel, which may raise questions about the generalizability of our results to other populations and regions affected by armed conflict. However, it is worth noting that by examining a specific context, we were able to gain in-depth understanding and provide valuable insights into the challenges faced by this particular population.

Another potential limitation is the reliance on available data, which could introduce selection bias and limitations related to retrospective data. However, we took great care in analyzing and interpreting the data, ensuring transparency and acknowledging the inherent limitations of retrospective analysis. By addressing these limitations and providing thorough discussions on the data collection process, we aim to mitigate any concerns related to the reliability and validity of our findings.

Furthermore, as an observational study, our research design does not establish causal relationships between conflict-related disruptions in routine care and the observed outcomes. However, by carefully controlling for confounding variables and considering alternative explanations, we have provided compelling evidence of the significant impact of armed conflict on dementia care. We believe that our study contributes to the existing body of knowledge and serves as a foundation for future research endeavors.

Despite these limitations, our findings highlight the rapid changes in dementia care paradigms during armed conflict, emphasizing the urgent need for tailored strategies and interventions. By acknowledging the limitations of our study and proposing avenues for future research, we hope to stimulate further investigations that can confirm and expand on our findings.

## Conclusion

Our study provides valuable insights into the challenges of dementia care during armed conflict, specifically focusing on the elderly population in Israel. The findings highlight the profound impact of conflict on routine care paradigms and emphasize the importance of robust and adaptable healthcare systems capable of maintaining care continuity in such challenging circumstances.

The dynamic changes observed in care paradigms during the conflict underscore the need for preparedness and flexibility in healthcare delivery. It also emphasizes the significance of further research, particularly larger-scale, multi-country studies that can compare crisis responses and inform best practices in dementia care during armed conflicts.

While our study is observational and cannot establish causal relationships, it serves as a steppingstone for future research and policymaking in dementia care during armed conflicts. The lessons learned from this study can contribute to the development of more resilient healthcare systems that can effectively serve vulnerable populations, such as the elderly with dementia, even in the midst of armed conflict.

In conclusion, our study sheds light on the complexities of dementia care during armed conflict, providing guidance for future research and policy development. By integrating these insights, we can work towards improving the quality of care and support for individuals with dementia in conflict-affected regions.

## Supplementary Materials

The Supplementary data can be found online at: www.aginganddisease.org/EN/10.14336/AD.2024.0432.
