# Supplementary material for: Conflict-Related Breakdowns in Routine Medical Care - Longitudinal Outcomes for Civilians with Pre-Existing Cognitive Impairment
Source: Aging Dis. 2024 May 31;16(3):1586–97. doi: 10.14336/AD.2024.0432 (PMC12096909; doi:10.14336/AD.2024.0432)
Supplement: Supplementary file 1 — The Supplementary data can be found online at: www.aginganddisease.org/EN/10.14336/AD.2024.0432. [file AD-16-3-1586-s.pdf]

SUPPLEMENTARY DATA

Conflict-Related Breakdowns in Routine Medical Care - Longitudinal Outcomes for Civilians with Pre-Existing Cognitive Impairment

Zorian Radomyslsky, Sara Kivity, Yaniv Alon, Mor Saban

© 2024. Radomyslsk Zet al. Published online at http://www.aginganddisease.org/EN/10.14336/AD.2024.0432

SUPPLEMENTARY DATA

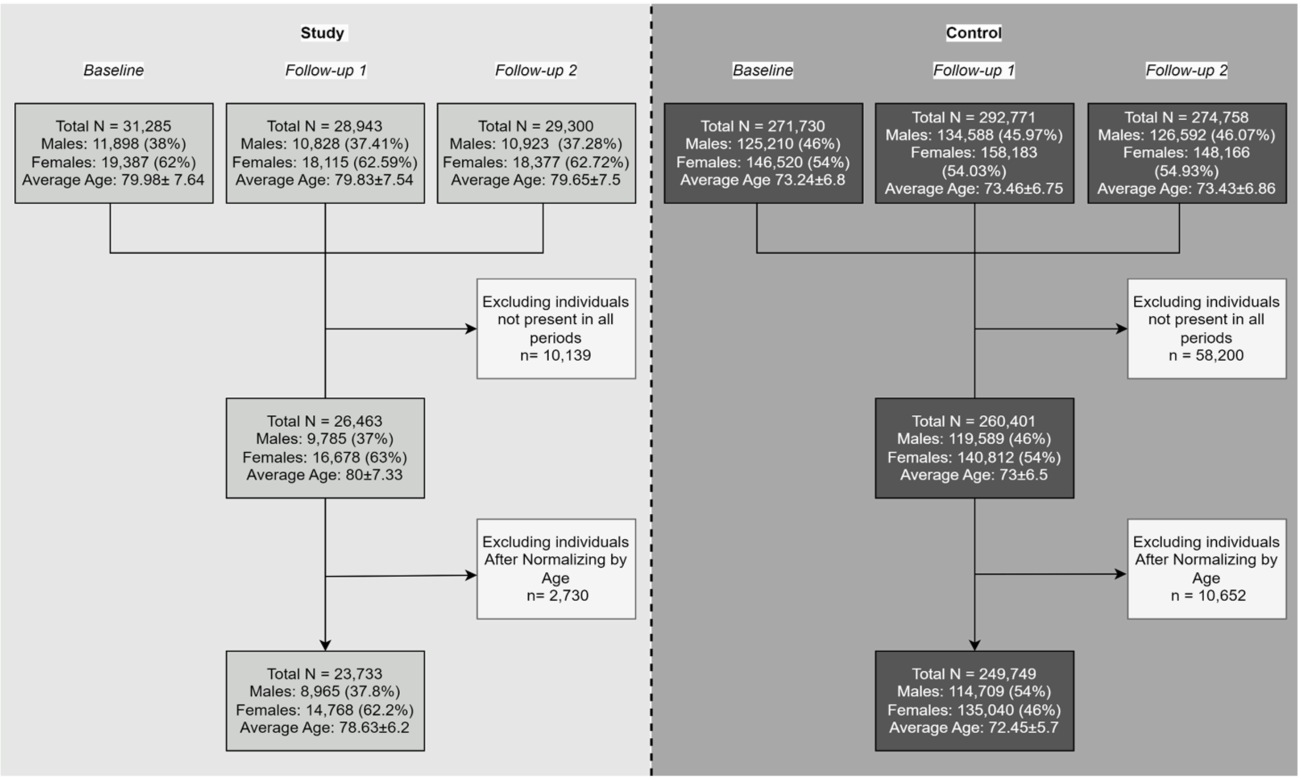

Supplementary Figure 1. Individual Selection and Exclusion Flow Chart from Baseline to Second Follow-Up. The flow chart delineates the process of selecting and excluding individuals for a comparative study between Study and Control groups, assessed at three key time points: the Baseline (prior to October 7, 2023), the First Follow-up (on December 31, 2023), and the Second Follow-up (on February 27, 2024). Each group assessed on the two subsequent dates, with individuals do not present at all time points being excluded. After each exclusion step, the remaining individuals were normalized by age. The final counts and demographic details at the end of this process are presented for both the Study and Control groups.

Supplementary Table 1. GEE Analysis Results Impact of Time and Group Status on Elderly Different Health Conditions.

Condition	Variable	Coefficient	StdErr	z	P>|z|	CI_low	CI_high
Dementia	Intercept	-36.640	0.008	-4729.535	p<0.001	-36.655	-36.625
	Period (Follow-up 1 vs. Baseline)	-2.567	0.002	-1146.618	p<0.001	-2.571	-2.562
	Period (Follow-up 2 vs. Baseline)	0.849	0.003	288.186	p<0.001	0.843	0.855
	Group (Study vs. Control)	35.324	0.008	4559.701	p<0.001	35.309	35.340
	Period*Group (Follow-up 1*Study)	2.531	0.002	1130.818	p<0.001	2.527	2.536
	Period*Group (Follow-up 2*Study)	-0.888	0.003	-301.518	p<0.001	-0.894	-0.883
Depression	Intercept	-2.317	0.007	-340.901	p<0.001	-2.330	-2.304
	Period (Follow-up 1 vs. Baseline)	-0.403	0.005	-74.936	p<0.001	-0.413	-0.392
	Period (Follow-up 2 vs. Baseline)	-0.374	0.005	-70.369	p<0.001	-0.385	-0.364
	Group (Study vs. Control)	1.378	0.016	88.170	p<0.001	1.348	1.409
	Period*Group (Follow-up 1*Study)	-0.078	0.013	-6.130	p<0.001	-0.103	-0.053
	Period*Group (Follow-up 2*Study)	-0.147	0.013	-11.062	p<0.001	-0.173	-0.121
Blood pressure	Intercept	0.587	0.004	144.758	p<0.001	0.579	0.595
	Period (Follow-up 1 vs. Baseline)	-0.105	0.002	-61.729	p<0.001	-0.108	-0.101
	Period (Follow-up 2 vs. Baseline)	-0.023	0.001	-27.764	p<0.001	-0.024	-0.021

© 2024. Radomyslsk Zet al. Published online at http://www.aginganddisease.org/EN/10.14336/AD.2024.0432

SUPPLEMENTARY DATA

	Group (Study vs. Control)	0.632	0.016	40.481	p<0.001	0.601	0.663
	Period*Group (Follow-up 1*Study)	0.057	0.006	10.288	p<0.001	0.046	0.068
	Period*Group (Follow-up 2*Study)	-0.040	0.006	-6.250	p<0.001	-0.053	-0.028
Chronic obstructive pulmonary disease (COPD)	Intercept	-2.461	0.007	-341.001	p<0.001	-2.476	-2.447
	Period (Follow-up 1 vs. Baseline)	-0.091	0.003	-32.857	p<0.001	-0.097	-0.086
	Period (Follow-up 2 vs. Baseline)	-0.037	0.002	-16.662	p<0.001	-0.041	-0.032
	Group (Study vs. Control)	0.243	0.022	10.811	p<0.001	0.199	0.287
	Period*Group (Follow-up 1*Study)	0.075	0.007	10.932	p<0.001	0.061	0.088
	Period*Group (Follow-up 2*Study)	0.017	0.009	1.986	p<0.05	0.000	0.034
Diabetes mellitus (DM)	Intercept	-0.840	0.004	-198.459	p<0.001	-0.848	-0.832
	Period (Follow-up 1 vs. Baseline)	-0.065	0.002	-39.159	p<0.001	-0.068	-0.061
	Period (Follow-up 2 vs. Baseline)	0.044	0.002	23.794	p<0.001	0.040	0.047
	Group (Study vs. Control)	0.340	0.014	24.776	p<0.001	0.313	0.367
	Period*Group (Follow-up 1*Study)	0.047	0.004	10.562	p<0.001	0.039	0.056
	Period*Group (Follow-up 2*Study)	0.000	0.007	-0.023	0.98	-0.014	0.014
Immune suppression	Intercept	-1.591	0.005	-307.050	p<0.001	-1.601	-1.580
	Period (Follow-up 1 vs. Baseline)	-0.133	0.002	-56.269	p<0.001	-0.138	-0.129
	Period (Follow-up 2 vs. Baseline)	-1.105	0.007	-164.481	p<0.001	-1.118	-1.092
	Group (Study vs. Control)	0.157	0.017	9.281	p<0.001	0.123	0.190
	Period*Group (Follow-up 1*Study)	0.121	0.006	20.136	p<0.001	0.109	0.133
	Period*Group (Follow-up 2*Study)	0.181	0.019	9.538	p<0.001	0.144	0.218
Obesity	Intercept	-0.960	0.004	-221.166	p<0.001	-0.969	-0.952
	Period (Follow-up 1 vs. Baseline)	0.023	0.001	16.044	p<0.001	0.021	0.026
	Period (Follow-up 2 vs. Baseline)	0.871	0.003	250.168	p<0.001	0.864	0.877
	Group (Study vs. Control)	0.118	0.014	8.159	p<0.001	0.090	0.146
	Period*Group (Follow-up 1*Study)	-0.035	0.004	-7.981	p<0.001	-0.044	-0.027
	Period*Group (Follow-up 2*Study)	0.140	0.013	10.879	p<0.001	0.115	0.166
Hip Fracture	Intercept	-2.580	0.008	-339.887	p<0.001	-2.595	-2.565
	Period (Follow-up 1 vs. Baseline)	-1.447	0.014	-101.145	p<0.001	-1.475	-1.419
	Period (Follow-up 2 vs. Baseline)	-1.561	0.016	-95.842	p<0.001	-1.593	-1.529
	Group (Study vs. Control)	0.419	0.022	18.941	p<0.001	0.376	0.463
	Period*Group (Follow-up 1*Study)	-0.100	0.043	-2.322	p<0.05	-0.184	-0.016
	Period*Group (Follow-up 2*Study)	-0.053	0.046	-1.164	0.24	-0.143	0.036
**Antipsychotic usage**	Intercept	-3.211	0.010	-319.016	p<0.001	-3.230	-3.191
	Period (Follow-up 1 vs. Baseline)	-0.630	0.011	-57.931	p<0.001	-0.651	-0.608
	Period (Follow-up 2 vs. Baseline)	-0.728	0.012	-60.202	p<0.001	-0.752	-0.705
	Group (Study vs. Control)	2.036	0.018	113.249	p<0.001	2.001	2.072
	Period*Group (Follow-up 1*Study)	0.167	0.017	9.969	p<0.001	0.134	0.200
	Period*Group (Follow-up 2*Study)	0.159	0.019	8.355	p<0.001	0.122	0.196
**Antipsychotic prescriptions**	Intercept	-3.376	0.011	-310.726	p<0.001	-3.397	-3.354
	Period (Follow-up 1 vs. Baseline)	-0.597	0.012	-51.070	p<0.001	-0.620	-0.574
	Period (Follow-up 2 vs. Baseline)	-0.775	0.014	-56.788	p<0.001	-0.802	-0.748
	Group (Study vs. Control)	1.994	0.019	104.018	p<0.001	1.957	2.032

© 2024. Radomyslsk Zet al. Published online at http://www.aginganddisease.org/EN/10.14336/AD.2024.0432

	Period*Group (Follow-up 1*Study)	0.065	0.019	3.414	p<0.001	0.028	0.103
	Period*Group (Follow-up 2*Study)	0.063	0.022	2.807	p<0.01	0.019	0.107
**Antidepressant Usage**	Intercept	-1.278	0.005	-271.636	p<0.001	-1.287	-1.269
	Period (Follow-up 1 vs. Baseline)	-0.514	0.004	-126.569	p<0.001	-0.522	-0.506
	Period (Follow-up 2 vs. Baseline)	-0.640	0.005	-138.915	p<0.001	-0.649	-0.631
	Group (Study vs. Control)	1.292	0.014	95.669	p<0.001	1.265	1.318
	Period*Group (Follow-up 1*Study)	-0.110	0.012	-9.475	p<0.001	-0.133	-0.087
	Period*Group (Follow-up 2*Study)	-0.157	0.013	-11.996	p<0.001	-0.183	-0.131
**Antidepressant prescriptions**	Intercept	-1.260	0.005	-269.135	p<0.001	-1.269	-1.251
	Period (Follow-up 1 vs. Baseline)	-0.517	0.004	-124.718	p<0.001	-0.525	-0.509
	Period (Follow-up 2 vs. Baseline)	-0.699	0.005	-144.654	p<0.001	-0.709	-0.690
	Group (Study vs. Control)	1.194	0.014	88.447	p<0.001	1.168	1.220
	Period*Group (Follow-up 1*Study)	-0.129	0.012	-10.740	p<0.001	-0.153	-0.106
	Period*Group (Follow-up 2*Study)	-0.203	0.014	-14.552	p<0.001	-0.231	-0.176
**Eligible for Home Treatments**	Intercept	-4.327	0.017	-252.720	p<0.001	-4.360	-4.293
	Period (Follow-up 1 vs. Baseline)	-0.385	0.012	-33.271	p<0.001	-0.407	-0.362
	Period (Follow-up 2 vs. Baseline)	-0.361	0.016	-22.884	p<0.001	-0.391	-0.330
	Group (Study vs. Control)	2.019	0.028	72.306	p<0.001	1.964	2.074
	Period*Group (Follow-up 1*Study)	0.192	0.018	10.729	p<0.001	0.157	0.227
	Period*Group (Follow-up 2*Study)	0.151	0.023	6.609	p<0.001	0.106	0.195

Summarized results from a Generalized Estimating Equations (GEE) analysis evaluated the impact of time periods and group status on various health conditions within an elderly population. The analysis includes coefficients, standard errors, z-scores, p-values, and confidence intervals for each variable in the model. Assessed conditions encompass Dementia, Depression, Chronic Obstructive Pulmonary Disease (COPD), Diabetes Mellitus (DM), Immune Suppression, Obesity, Hip Fracture, Eligibility for Home Treatments, and usage/prescriptions of Antidepressant and Antipsychotic medications. Variables indicate comparisons between time periods and differences between the control and study groups as follows: Intercept: The average outcome at the start of the study for the control group. Period (Follow-up 1 vs. Baseline): The change in the outcome from baseline to the first follow-up. Period (Follow-up 2 vs. Baseline): The change in the outcome from baseline to the second follow-up. Group (Study vs. Control): The difference in outcome between the study group and control group at baseline. Period*Group (Follow-up 1*Study): The difference in the first follow-up changes between the study and control groups. Period*Group (Follow-up 2*Study): The difference in the second follow-up changes between the study and control groups.

Supplementary Table 2. Associations Between Depression, Antidepressant Use, and Immune Suppression: Mixed Linear Model Analysis.

**Model:**	**MixedLM**	**Dependent Variable:**	**Immune suppression**			
**No. Observations:**	876656	Method:	REML			
**No. Groups:**	2	Scale:	0.1120			
**Min. group size:**	77373	Log-Likelihood:	-284209.2698			
**Max. group size:**	799283	Converged:	Yes			
**Mean group size:**	438328.0					
**index**	**Coef.**	**Std.Err.**	**z**	**P>|z|**	**[0.025**	**0.975]**
**Intercept**	0.175	0.237	0.742	0.458	-0.288	0.639
**Time**	-0.050	0.237	-0.211	0.833	-0.514	0.414
**Depression**	0.048	0.002	24.244	0.000	0.044	0.052
**Antidepressant adherence**	0.051	0.001	47.799	0.000	0.049	0.053
**Depression:Antidepressant adherence**	-0.045	0.003	-16.490	0.000	-0.050	-0.039
**Group Var**	0.112					
**Group x Time Cov**	0.000					

SUPPLEMENTARY DATA

**Time Var**	0.112					

Results of a mixed linear model regression assessing the effects of depression diagnosis and antidepressant use on immune suppression, based on 876,656 observations.

© 2024. Radomyslsk Zet al. Published online at http://www.aginganddisease.org/EN/10.14336/AD.2024.0432
